# Surface-Enhanced Raman Scattering Study of the Product-Selectivity of Plasmon-Driven Reactions of p‑Nitrothiophenol in Silver Nanowires

**DOI:** 10.1021/acsomega.5c08431

**Published:** 2025-10-01

**Authors:** Cintia R. Petroni, Jonnatan J. Santos, Douglas S. Lopes, Daniele C. Ferreira, Gustavo F. S. Andrade, Paola Corio

**Affiliations:** † Federal Institute of São Paulo, Suzano, São Paulo 08673010, Brazil; ‡ Department of Fundamental Chemistry, Institute of Chemistry, 28133 University of Sao Paulo, São Paulo 05508000, Brazil; § Laboratório de Nanoestruturas Plasmônicas, Núcleo de Espectroscopia e Estrutura Molecular, Departamento de Química, 28113 Universidade Federal de Juiz de Fora, Juiz De Fora 36036-900, Brazil

## Abstract

Nanoplasmonic photocatalysis shows a huge potential for efficient light energy harvesting by leveraging the unique and peculiar properties of plasmonic metallic nanostructures (usually Ag or Au) to drive light-induced chemical reactions. Plasmonic catalysis of the coupling reaction of *p*-nitrothiophenol (PNTP) over silver nanowires (AgNWs) and -quasi-spherical silver nanoparticles (AgNPs) has been intensively investigated by in situ surface-enhanced Raman scattering (SERS). The reduction of PNTP is often used as a model system in plasmonic photocatalysis basic science since the conversion of the nitro group into an amino (*p*-aminothiophenol, PATP) or an azo (4,4′-dimercaptoazobenzene, DMAB) group is a well-documented reaction that can occur through a wide variety of chemical processes, allowing for the study of fundamental reaction mechanisms. However, the reduction of PNTP to PATP rarely occurs in the absence of a strong reducing species, such as H_2_ or NaBH_4_. The control and understanding of the precise molecular mechanisms of plasmonic catalysis in different chemical reactions involving different metallic nanostructures are an object of intense interest for possible applications. In this context, SERS is a powerful tool since it enables in situ tracking of the catalytic reactions because it combines the advantages of high chemical specificity (vibrational Raman scattering), high sensitivity, and surface selectivity. Our study demonstrated selectivity in the surface reactions of PNTP on AgNWs (PATP and DMAB) and AgNPs (DMAB). For the first time, the reduction of PNTP to PATP in the presence of AgNWs in air without the use of any strong reducing agent was observed. In addition, the dependence of the reaction on exciting radiation and on laser power density was investigated to control the selectivity of the reaction mechanism on AgNWs. Finally, the crucial function of the nanoparticle shape and anisotropy in the reaction mechanism is highlighted.

## Graphical abstract

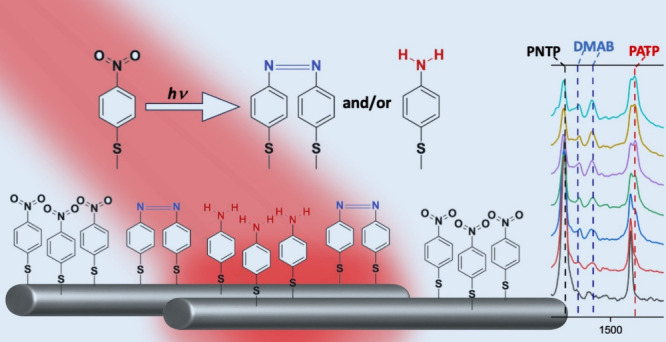

## Introduction

Metallic plasmonic nanoparticles (NPs) exhibit unique optical properties due to the possibility of exciting the collective oscillation of surface electrons, known as localized surface plasmon resonance (LSPR). This plasmonic behavior is closely dependent on the chemical nature, size, and shape, among other properties, of the NPs. Tuning and controlling the plasmonic resonance by varying the size and shape of the NPs can be highly beneficial in expanding their application fields, particularly in areas such as sensing, light and energy harvesting, biomedicine, and catalysis.^1,2^ This is because, while spherical nanoparticles exhibit plasmon resonance over a relatively narrow wavelength range, anisotropic nanoparticles offer more options for controlling the plasmonic resonance wavelength, from the visible to the mid-infrared, depending on the morphology and aspect ratio of the NPs.^1^ Such LSPR tuning can be crucial when employing techniques such as surface-enhanced Raman spectroscopy (SERS), which utilize nanostructured plasmonic metal substrates.^3^

The surface plasmon resonance of silver and gold nanoparticles significantly enhances the efficiency of a myriad of photocatalytic processes, ranging from water splitting reaction,^4^ reduction of CO_2_ into hydrocarbon fuels,^5^ and C–C coupling^6^ reactions, among others.^6^ One potential application of the SERS technique is the in situ monitoring of chemical reactions mediated by visible light on the surface of plasmonic NPs. These reactions are part of so-called plasmonic photocatalysis, a growing field of interest, as the employed nanocatalysts allow for lower temperatures and increased reaction efficiencies.^7^ Several mechanisms have been proposed for plasmon-driven reactions, explained by the excitation of LSPR due to strong light–matter interaction, resulting in enhanced local electromagnetic fields in the proximity of the metal surface.^7^ Once excited, LSPR can decay either radiatively through photon re-emission or nonradiatively via excitation of high-energy electron–hole pairs, known as hot electron–hole pairs. These hot electrons may be injected into unoccupied orbitals of adsorbed molecules to initiate various bond-breaking and bond-forming processes, thus being surface plasmon-induced.^8^ Anisotropic nanoparticles, such as nanocubes (with edges) and nanocones or nanostars (with tips), exhibit significantly stronger local electromagnetic field enhancements than spherical nanoparticles of similar size^9^ and can concentrate the incident radiation with improved efficiency, thereby significantly increasing hot electron generation by the nanostructure.^7^

In this work, catalytic and SERS-responsive functionalities of silver nanowires (AgNWs) and quasi-spherical silver nanoparticles (AgNPs) were achieved for the in situ photochemical reactions of p-nitrothiophenol (PNTP). This is a well-studied model reaction in plasmon-mediated chemical processes investigated by SERS, as the reduction of p-nitrothiophenol (PNTP) can produce 4,4′-dimercaptoazobenzene (DMAB) or p-aminothiophenol (PATP). In addition, PNTP is a strong Raman scatterer that forms stable monolayers on metal surfaces due to its thiol group, which allows SERS monitoring of the reaction progress with high sensitivity. The above-mentioned reactions can proceed through several pathways, including the conversion of PNTP to PATP, the conversion of PNTP to DMAB, the conversion of DMAB to PATP, the conversion of PATP to DMAB, or a combination of the mentioned reactions.^10,11^ However, although PNTP can be transformed into DMAB via plasmonic catalysis, its reduction to PATP rarely occurs in the absence of a reducing chemical species, such as H_2_ or NaBH_4_.^12,13^

## Results and Discussion

Figure 1 shows the extinction spectra and scanning electron microscopy (SEM) images of the nanostructures used in this work. Predominantly quasi-spherical particles (Figure 1B) with an average diameter of ∼100 nm (Figure S1) and wires (Figure 1C) with major axes in the micrometer range and minor axes of ∼140 nm (Figure S1) can be observed in the SEM micrographs of the AgNPs and AgNWs, respectively. The extinction spectra of AgNPs, presented in Figure 1A, a band at 482 nm appears, characteristic of silver nanoparticles with a diameter close to 100 nm.^14^ For the AgNWs, on the other hand, the extinction spectra in Figure 1A present two bands near 400 nm, both related to different LSPRs from this anisotropic Ag nanostructure. The band peaking at 406 nm is attributed to the dipolar LSPR mode of the AgNWs, while the 359 nm band corresponds to the quadrupolar LSPR mode.^15^

**Figure 1:**
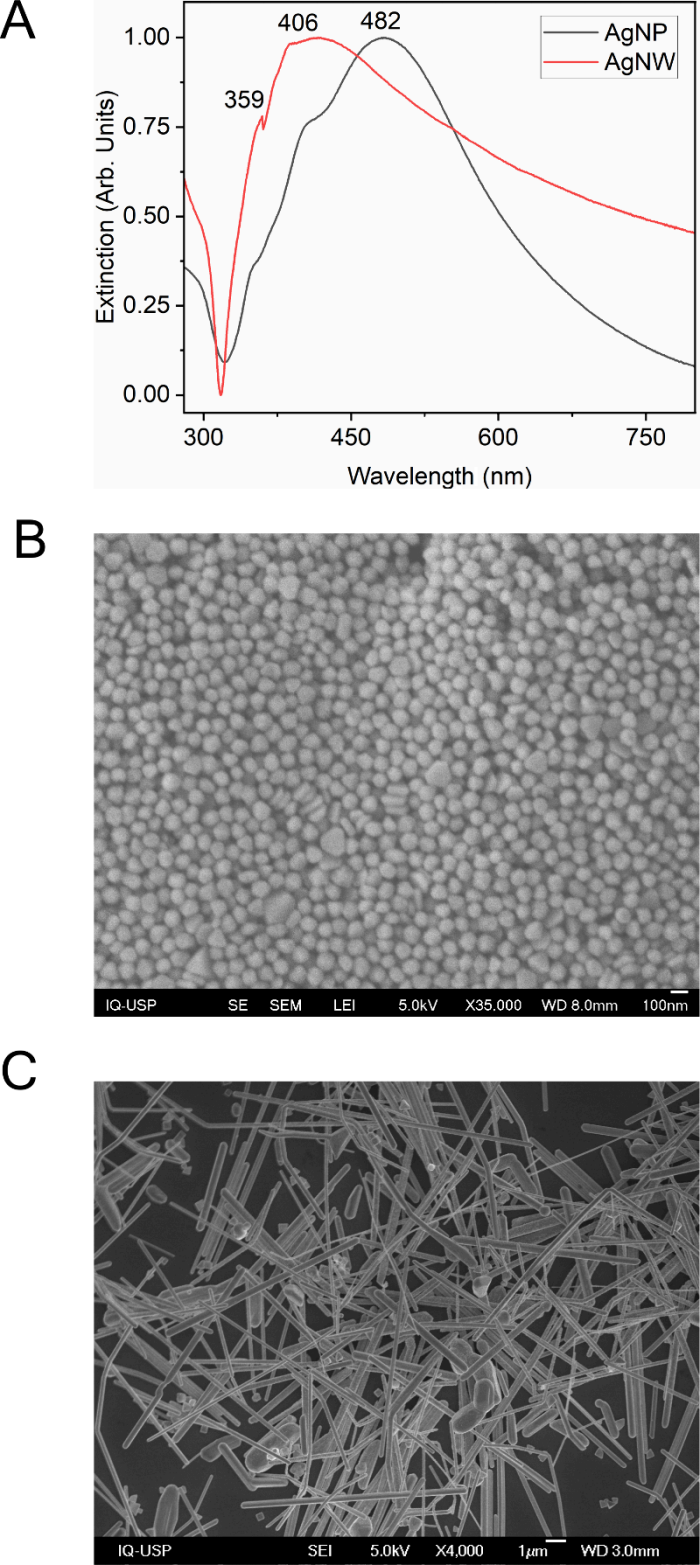
Extinction spectra of AgNPs and AgNWs (A) and SEM images of AgNPs (B) and AgNWs (C).

Thiols such as PNTP and PATP have been widely used in plasmon-driven model reactions monitored by surface-enhanced techniques, such as SERS and TERS.^16–21^ Among the reactions involving these species, there are the coupling of PNTP to form DMAB and its reduction to PATP, both of which are addressed in the present study. The surface reactions of PNTP adsorbed on Ag nanostructures were monitored by SERS, with spectra recorded at the same spot while increasing the laser power at 633 nm and using an acquisition time of 1 s for each spectrum. These spectra are shown in Figure 2A,B, where the emergence and intensification of bands at 1144, 1388, and 1437 cm^–^^1^ (attributed to DMAB) can be observed with increasing laser power, while the 1336 cm^–^^1^ band (assigned to the symmetric NO_2_ stretch – νsNO_2_ – of PNTP) decreases, indicating PNTP consumption and concomitant DMAB formation for both AgNPs and AgNWs. Surprisingly, in the presence of the wires, starting from 3 mW, a new band at 1590 cm^–^^1^, attributed to PATP, also emerges, even in the absence of a reducing agent (Figure 2B). The assignments of the main bands of these molecules are presented in Table S1, and the corresponding SERS spectra are shown in Figure S2.

**Figure 2:**
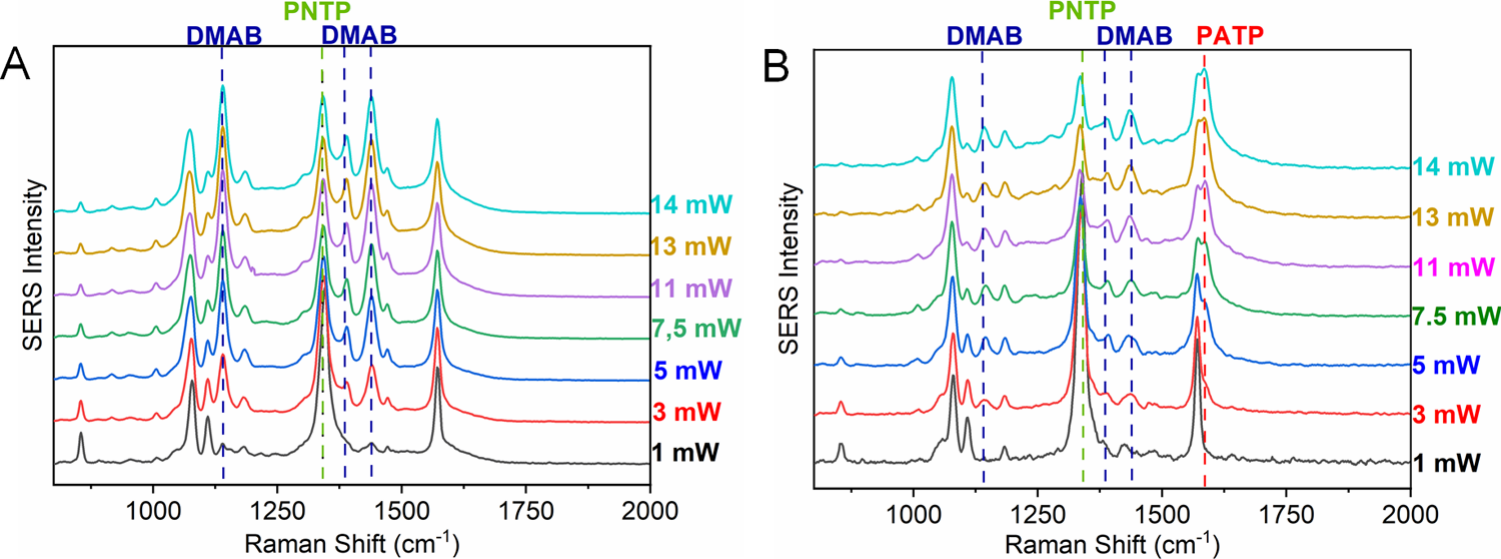
Laser power tests of PNTP adsorbed on (A) AgNP and (B) AgNW in air, with laser power ranging from 1 to 14 mW and an acquisition time of 1 s. λ_0_ = 633 nm. All spectra were normalized to the 1081 cm^–1^ band.

The coupling reaction of PNTP to form DMAB on the NP surfaces showed dependence on the incident power density—typical of plasmon-driven reactions. Catalytic experiments as a function of power showed a linear relationship between the 1438 cm^–^^1^ band (attributed to the NN azo stretch – νNN – in DMAB) and the laser power, with monotonic band growth, as can be observed in Figure 3A. This linearity at low laser power values suggests the predominance of nonthermal reaction mechanisms, dominated by the transfer of excited charge carriers (hot electrons and holes),^22^ in agreement with studies reported for the PNTP coupling reaction.^16,21,23^ In the experiments using AgNPs, a higher yield in the formation of DMAB from PNTP can also be observed, as indicated by the slope of the line, compared to experiments using AgNWs (Figure 3A). However, regarding the consumption of PNTP, based on the decrease in intensity of the band at 1338 cm^–^^1^ (ν_s_NO_2_), a more pronounced decrease was observed in the presence of AgNWs (Figure 3B), even though it does not lead to a higher formation of DMAB. These results, together with the appearance of the band at 1590 cm^–^^1^ (Figure 2B), suggest that PNTP was not only fully converted into DMAB but also into PATP in the presence of AgNWs. Kinetic data from time-dependent SERS spectra, as well as the relative intensities of the 1438 cm^–^^1^ band (DMAB) as a function of irradiation time, were obtained (Figure S3). From the slope of the linear portion at the initial stages of radiation exposure in the SERS intensity-time relationship, the DMAB formation rate on AgNW was determined. It is important to note that in time-dependent catalytic experiments, it is necessary to ensure that there is no product at the beginning of the reaction, and it should be possible to calculate the conversion rate from the initial linear portion. For this reason, a lower irradiation power was used compared to power-dependent experiments.

**Figure 3:**
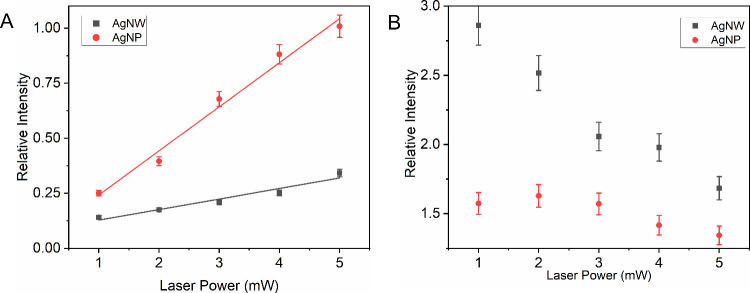
Normalized intensity of the (A) 1438 cm^–^^1^ band (νNN in DMAB) and (B) 1338 cm^–1^ band (ν_s_NO_2_) as a function of laser power. λ_0_ = 633 nm. The normalization was to the 1081 cm^–1^ band.

Catalytic hydrogenation of PNTP to PATP typically requires strong chemical reducing agents such as sodium borohydride^24^ or hydrogen gas,^25^ or plasmon-mediated reactions in acidic media.^26,27^ Thus, experiments were carried out at pH 2 (using aqueous HCl) for both AgNPs and AgNWs, as PATP formation has been reported under these conditions.^26,27^ As expected, Figure 4A,B shows the emergence of the 1590 cm^–^^1^ PATP band (assigned to the νCC + δNH_2_ PATP characteristic mode^28^), along with DMAB bands, for both AgNPs and AgNWs nanostructures. Catalytic experiments as a function of power showed a dependent relationship between the 1590 cm^–^^1^ band and the incident radiation power, although with a higher rate of increase in the presence of AgNW compared to that of AgNP (Figure S4).

**Figure 4:**
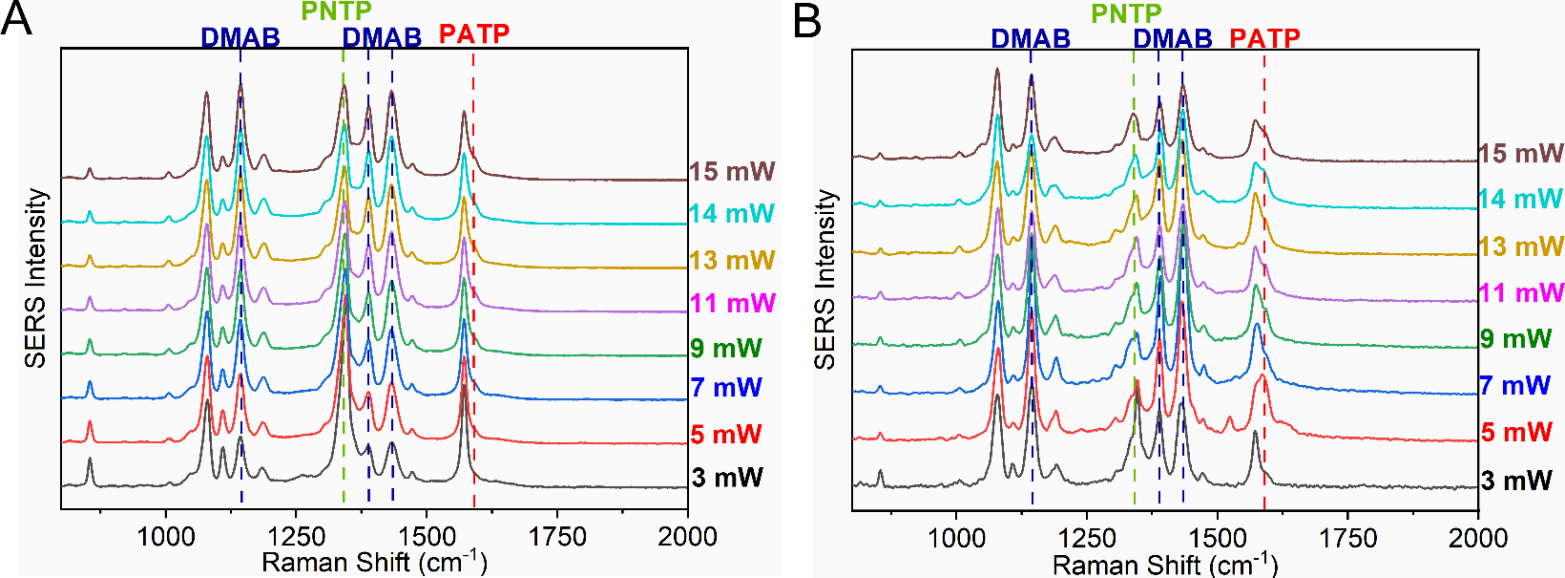
Effect of laser power at pH 2 for PNTP adsorbed on (A) AgNPs and (B) AgNWs, with power ranging from 3.0 to 15.0 mW, acquisition time of 1 s, and λ_0_ = 633 nm. All spectra normalized to the 1081 cm^–^^1^ band.

As previously mentioned, LSPR may decay radiatively or nonradiatively via enhanced local electric fields, excited charge carrier generation, or localized plasmonic heating.^29,30^ Resonant excitation is crucial and directly linked to the NP’s size, morphology, composition, and surrounding dielectric environment.^31^ One way to tune LSPR is via plasmonic coupling of nanostructures, as closely spaced NPs can exhibit surface plasmon interactions, generating coupling modes with energies differing from those of individual NPs^32,33^ and highly sensitive to the nanogap dimension.^34^

Dark-field microscopy enabled the analysis of plasmonic resonance spectra of isolated and coupled AgNPs and AgNWs. Figure 5 shows the dark-field optical images and scattering spectra for AgNPs and AgNWs, with the white and red highlighted regions indicating the spot from which the corresponding spectra were obtained. For both spheres (Figure 5A,B) and wires (Figure 5C,D), significant scattering spectral differences were observed between isolated particles (black line) and coupled pairs (red line) for both nanostructures. Coupling of two AgNPs results in a new 688 nm band, while AgNWs coupling shifts the 488 nm band to 513 nm and introduces a new 634 nm band, resonant with the laser used in the catalytic tests. These results suggest that PATP formation from PNTP adsorbed on AgNWs in air is linked to the resonance between the coupled nanowire plasmon and the incident radiation wavelength, a phenomenon not observed with AgNPs.

**Figure 5:**
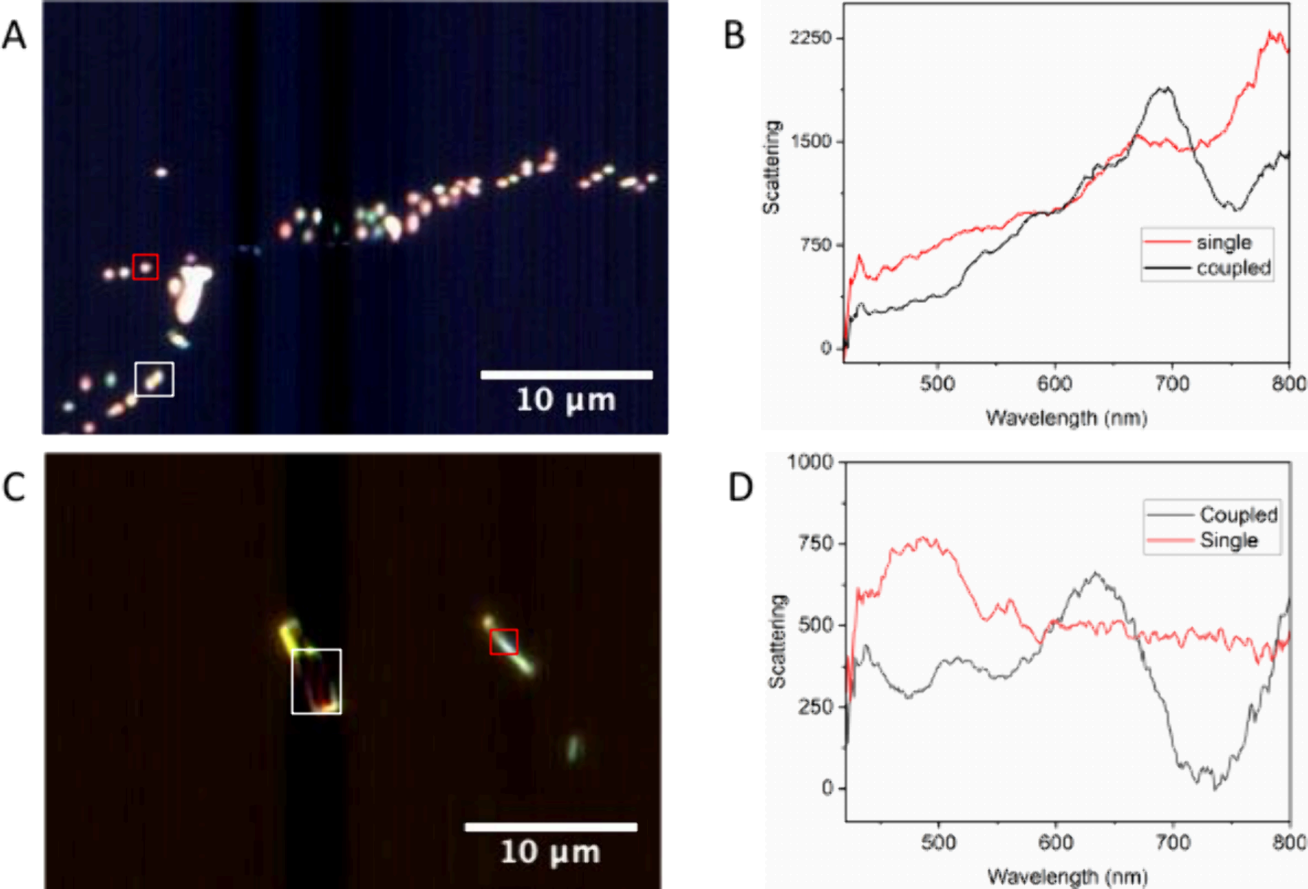
(A) Dark-field image of AgNPs and (B) their corresponding scattering spectra for a single particle and for two coupled particles. (C) Dark-field image of AgNWs and (D) their corresponding spectra for a single wire and for two coupled wires.

Based on the dark-field microscopy results suggesting plasmonic resonance between coupled AgNWs plasmon and the excitation wavelength, laser power tests were performed using 532 and 633 nm wavelengths on the same spot. The AgNWs sample was irradiated with 532 nm, followed by 633 nm light without changing the monitoring spot. The spectra are shown in Figure 6. At 532 nm (Figure 6A), a slight broadening at ∼1590 cm^–^^1^ ^25^ appears with increasing laser power, potentially linked to plasmon coupling at 513 nm. However, at 633 nm (Figure 6B), the 1590 cm^–^^1^ band clearly appears and intensifies with increasing laser power, indicating a correlation between the PNTP-to-PATP reduction in air and the incident radiation wavelength and power. Figure S5 shows the PATP/PNTP 1590/1338 cm^–1^ intensity ratios as a function of the laser power. An increase in the PATP/PNTP intensity ratios can be observed consistently with an increase in incident radiation power at a wavelength of 633 nm.

**Figure 6:**
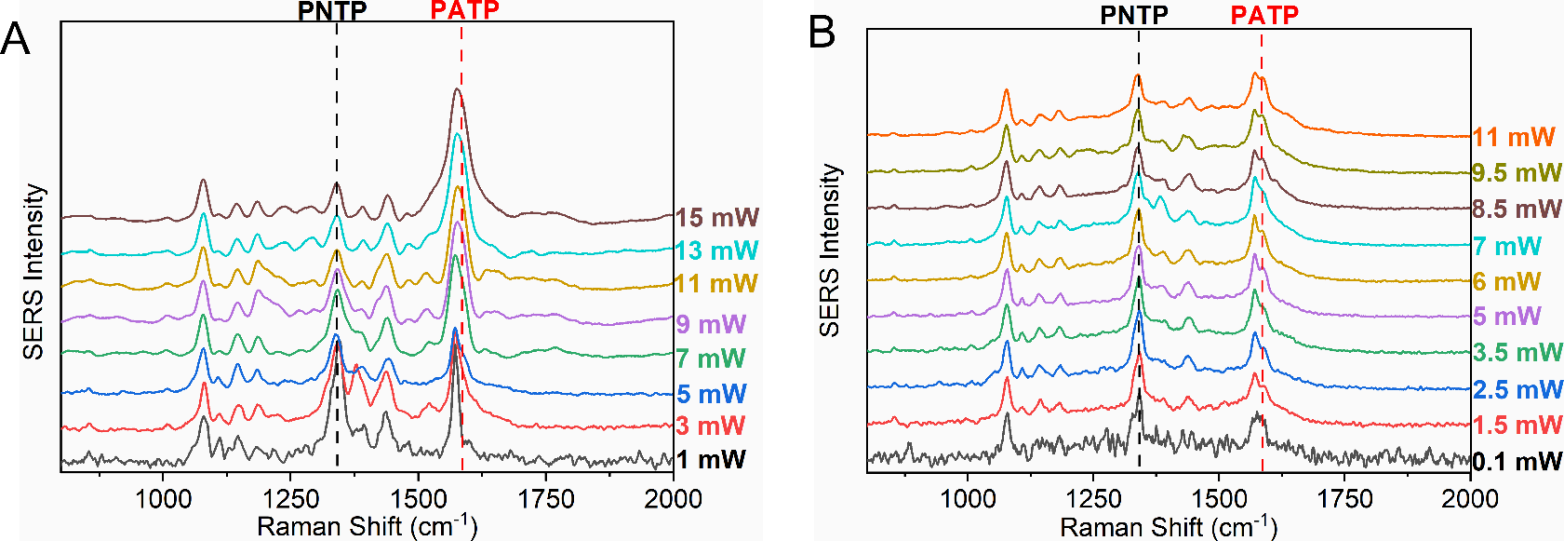
Laser power tests of PNTP adsorbed on AgNWs in air with 1 s acquisition. (A) λ_0_ = 532 nm, power from 1 to 15 mW; (B) λ_0_ = 633 nm, power from 0.1 to 11 mW. All spectra normalized to the 1081 cm^–^^1^ band.

To further investigate the correlation between PATP formation and laser power density, experiments were conducted using lenses with different numerical apertures (N.A.) while increasing the laser power. Spectra were obtained using 10× (N.A. 0.25, Figure 7A) and 50× (N.A. 0.8, Figure 7B) lenses, with 1 s irradiation time and an 1800 lines mm^–1^ grating. The 1590 cm^–1^ band appears starting at 2.75 × 10^8^ mW/cm^2^ for both objective lenses, and identical trends in the intensity of the 1590 cm^–^^1^ band as a function of power density (Figure 7C) were observed for both NAs, demonstrating a dependency of PNTP-to-PATP conversion on laser power density in air using AgNWs. A linear trend at low power density values was also observed in both cases.

**Figure 7:**
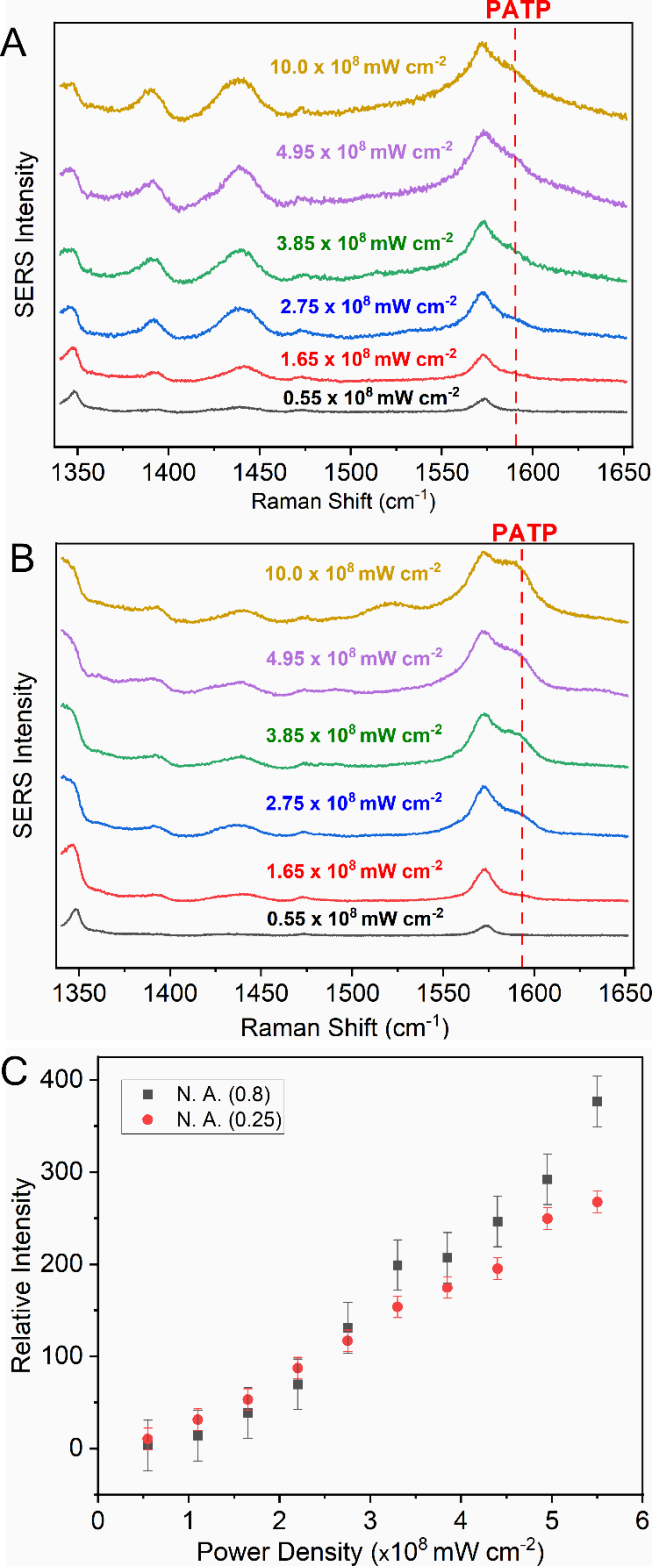
Laser power density tests with lenses of (A) N.A. 0.25 and (B) N.A. 0.8. (C) Intensity of the 1590 cm^–^^1^ band as a function of laser power density. All spectra were recorded at 633 nm, 1 s acquisition time, and 1800 g/mm grating.

## Conclusions

Our study demonstrated nanoparticle shape selectivity in the surface reactions of PNTP on AgNWs and AgNPs in air. The reaction of PNTP on AgNPs resulted in the preferential formation of DMAB, but on AgNWs, two products, DMAB and PATP—a reduction product—were detected using SERS spectroscopy. Differences in the dark-field scattering spectra of isolated and coupled AgNP and AgNW structures were observed and associated with the shape selectivity. Coupled AgNWs plasmons were resonant with the 633 nm laser, suggesting that this resonance enables PATP formation on wires, unlike with AgNPs. Laser power tests with different excitation wavelengths supported these findings, showing clear PATP signals on AgNWs at 633 nm and only a weak shoulder at 532 nm. The use of different laser spot sizes (based on objectives with different numerical apertures and magnifications) demonstrated a clear dependence of PATP formation on the laser power density. The present study may pave the way for engineering shape-selective plasmonic photocatalysts, thereby expanding the potential range of applications for these materials.

## Materials and Methods

### Materials and Instrumentation

AgNO_3_ (silver nitrate, anhydrous, *>*99%, Sigma-Aldrich), C_6_H_5_Na_3_O_7_.2H_2_O (trisodium citrate dihydrate, *>*99%, Sigma-Aldrich), O_2_NC_6_H_4_SH (4-nitrothiophenol, 80%, Sigma-Aldrich), (C_6_H_9_NO)_n_ (polyvinylpyrrolidone, *M*_w_ ∼ 55,000), HOCH_2_CH_2_OH (Ethylene glycol, >99%, Sigma-Aldrich), NaCl (sodium chloride, ≥99%), and C_76_H_52_O_46_ (tannic acid, Sigma-Aldrich) were used as purchased. Milli-Q water (18.2 MΩ cm) was used throughout all experiments. All glassware was washed with aqua regia and Milli-Q water prior to use.

Electronic extinction spectra were obtained from the colloidal Ag nanoparticle suspension in a UV–vis-NIR Shimadzu spectrometer, model UV-3101PC. The SEM images were obtained with a JEOL JSM-7401 scanning electron microscope using an acceleration voltage of 5 kV. Dark-field images were acquired using a CytoViva system, which employs an Olympus BX51 microscope modified to produce a hollow cone of light for dark-field illumination. A 100× objective lens with a numerical aperture (N.A.) of 1.00 was used. Hyperspectral images were captured using the same dark-field setup, with the addition of a transmission grating spectrophotometer operating in the 400–1000 nm spectral range. The system provided a spectral resolution of 2.8 nm and a spatial resolution of 128 nm per pixel. SERS spectra and catalytic laser-induced experiments were performed through an ultrahigh-throughput spectrometer (UHTS) from Witec, model 600-VIS, coupled to a Witec alpha300-R Raman confocal microscope. The laser beam, operating at a wavelength of 633 nm (He–Ne laser line) or 532 nm (diode-pumped solid-state), was focused on the sample using an objective lens (Zeiss Epiplan-Neofluar 10×/0.25 and 50×/0.8) and a true power module. The experiments were performed under ambient conditions.

### Synthesis of Silver Nanowires

The polyol synthesis is widely used for the preparation of anisotropic silver nanostructures, as it enables the preparation of nanowires, nanocubes, and other shapes in high yields.^35^ In a typical synthesis, 50.0 mg of PVP (polyvinylpyrrolidone, 55,000 g mol^–1^) was dissolved in 7.0 mL of ethylene glycol. To this mixture was added 300 μL of a 0.010 mol L^–1^ NaCl solution in ethylene glycol, and the reaction flask was placed in a silicone oil bath at 170 °C for approximately 1 h. After this period, 3 mL of a 0.10 mol L^–1^ AgNO_3_ solution in ethylene glycol was added with the aid of a peristaltic pump, and the mixture was maintained in an oil bath for approximately 1 h. The suspension was washed twice with water and redispersed before being stored.^36,37^

### Synthesis of Quasi-Spherical Silver Nanoparticles

Quasi-spherical silver nanoparticles (AgNPs) were obtained as previously published.^14^ AgNPs with an average diameter of approximately 90 nm were synthesized following a procedure adapted from Puntes et al.^17^ Initially, 75 mL of an aqueous solution containing 5 × 10^–^^3^ mol L^–^^1^ of sodium citrate and tannic acid was prepared using deionized water. The solution was heated under stirring in a three-neck round-bottomed flask until reflux was reached. Once boiling began, 1 mL of a 0.025 mol L^–^^1^ AgNO_3_ solution was added, resulting in an immediate color change to bright yellow, indicating the formation of nanoparticles. Following the synthesis of silver seeds, an initial growth step was carried out in the same flask. After the solution was allowed to cool to 90 °C, 0.10 mL of 0.025 mol L^–^^1^ sodium citrate, 0.25 mL of 0.25 mol L^–^^1^ tannic acid, and 0.25 μL of 0.025 mol L^–^^1^ AgNO_3_ were sequentially added to the reaction mixture, with approximately 1 min between each addition. This growth cycle was repeated six times. A secondary growth step was then performed. For this, 39 mL of the sample was diluted with 37 mL of Milli-Q water. The solution temperature was maintained at 90 °C, and sequential additions of 1 mL of 0.025 mol L^–^^1^ sodium citrate, 3 mL of 0.025 mol L^–^^1^ tannic acid, and 2 mL of 0.025 mol L^–^^1^ AgNO_3_ were made, again with ca. 1 min delay between additions. This process was repeated six times until the nanoparticles reached the desired diameter of ca. 90 nm.

### Catalytic Experiments

The functionalization of the SERS-active catalytic substrates was carried out by mixing 500 μL of the colloidal suspension of the nanostructures with 50 μL of a 1 × 10^–^^3^ mol L^–^^1^ ethanolic PNTP solution to obtain a final concentration of 1 × 10^–^^4^ mol L^–^^1^. This mixture was placed in an ultrasonic bath for 5 min and then centrifuged for 5 min, followed by a redispersion in water to the same volume. This procedure was repeated once more. The functionalized nanostructures were drop-cast onto a silicon wafer and dried in ambient air. They were then placed under a confocal Raman microscope, and the SERS signal was collected using 10× and 50× objective lenses.

## Supplementary Material



## Notes

Experimental section, histogram of nanoparticle diameter distribution, tentative assignment of the main bands in the SERS spectra, SERS spectra of PATP, PNTP, and DMAB adsorbed on the substrate, time-dependent SERS spectra of PATP, SERS intensity as a function of laser power, and additional references (PDF)
