# Supplementary material for: Surface-Enhanced Raman Scattering Study of the Product-Selectivity of Plasmon-Driven Reactions of p‑Nitrothiophenol in Silver Nanowires
Source: ACS Omega. 2025 Oct 1;10(41):49192–8. doi: 10.1021/acsomega.5c08431 (PMC12547744; doi:10.1021/acsomega.5c08431)
Supplement: Supplementary file 1 [file ao5c08431_si_001.pdf]

Surface-enhanced Raman scattering study of the product-selectivity of plasmon-driven reactions of p-nitrothiophenol in silver nanowires

Cintia R. Petroni[a,b]*, Jonnatan J. Santos*[b]**, Douglas S. Lopes*[b]*, Daniele C. Ferreira*[b]*,*

Gustavo F. S. Andrade[c]*, and Paola Corio*[b]

[a] Federal Institute of São Paulo, Suzano, São Paulo 08673010, Brazil;

[b] Department of Fundamental Chemistry, Institute of Chemistry, University of Sao Paulo, São Paulo 05508000, Brazil;

[c] Laboratório de Nanoestruturas Plasmônicas, Núcleo de Espectroscopia e Estrutura Molecular, Departamento de Química, Universidade Federal de Juiz de Fora, Juiz De Fora, 36036-900, Brazil;

*Email: jonnatan@iq.usp.br

KEYWORDS plasmonic silver nanoparticles, plasmon-enhanced catalysis, *in situ* SERS, darkfield and hyperspectral microscopy.

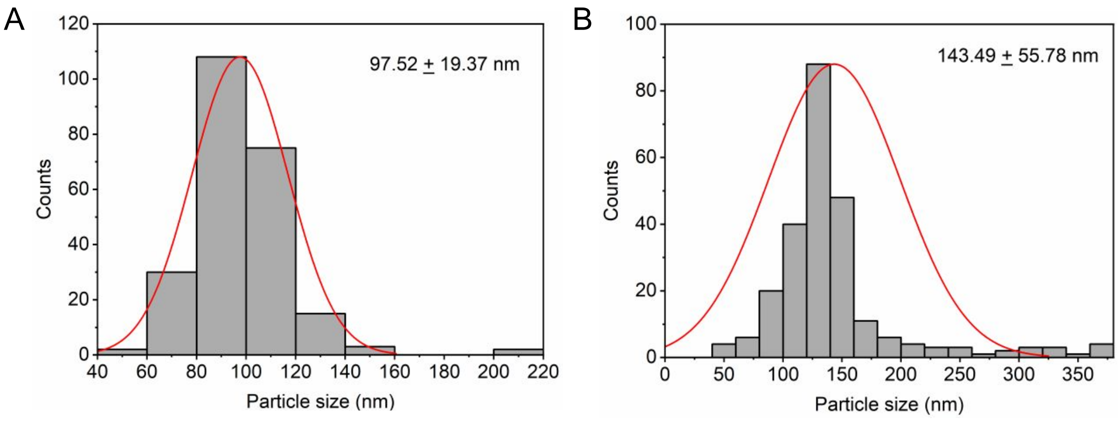

Figure S1. Diameter distribution of A) AgNPs and B) AgNW

Table S1. Tentative assignment of the main bands (wave number in cm^{-1}) in SERS spectra of the PATP, PNTP and DMAB on AgNW.

PATP	PNTP	DMAB	Assignments
-	854	-	$\beta(NO_2)$
1076	1081	1081	$\nu(CS)$
-	1107	-	$\nu(CH)$
-	-	1148	$\nu(CN) + \beta(CH)$
1180	-	-	$\beta(CH)$
-	1338	-	$\nu(NO_2)$
-	-	1394	$\nu(NN) + \nu(CC) + \beta(CH)$
-	-	1438	$\nu(NN) + \nu(CC) + \beta(CH)$
-	-	1472	$\nu(NN) + \nu(CC) + \beta(CH)$
1488			$\nu(CC) + \beta(CH)$
-	1574	1576	$\nu(CC)_{ring}$
1590	-	-	$\nu(CC)_{ring} + \beta(NH_2)$

ν = stretching mode and β = bending.

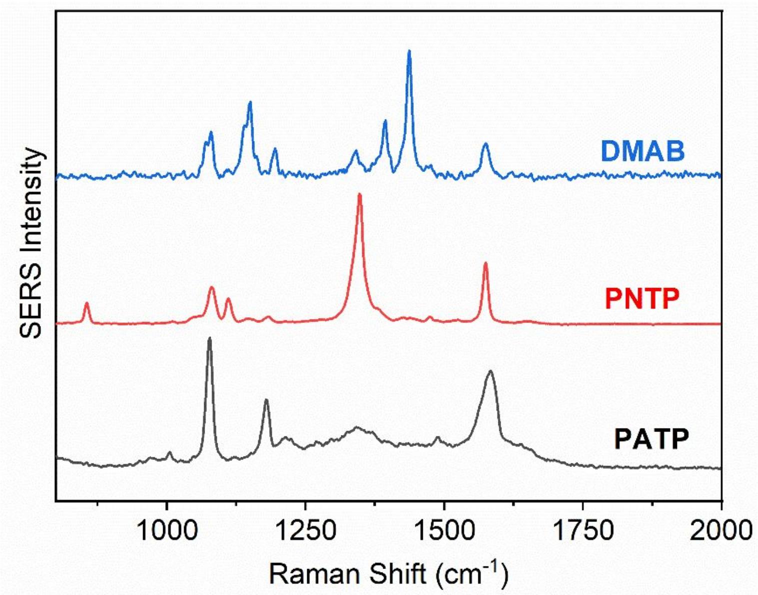

Figure S2. SERS spectra of PATP, PNTP and DMAB adsorbed on AgNW employing 0.1 mW for PATP, 0.5 mW for PNTP and 0.7 mW for DMAB as the 633 nm laser irradiation power. Spectra of PNTP and DMAB adsorbed on AgNW were obtained in air, but of PATP were obtained in HCl solution, pH 2. All spectra were normalized to the 1081 cm^{-1} band.

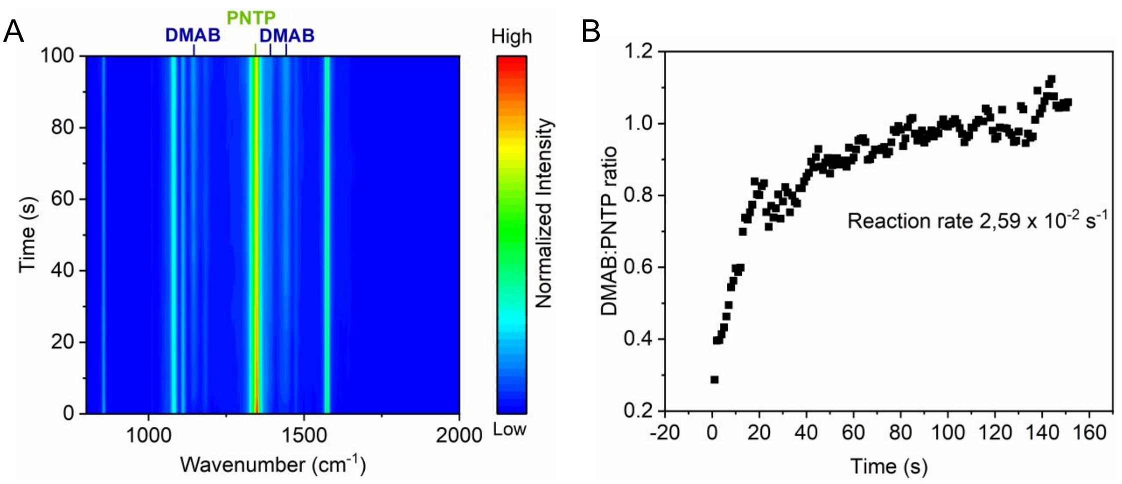

Figure S3. Time-dependent SERS spectra of A) PNTP adsorbed on AgNW in air employing 0.5 mW as the 633 nm laser irradiation power. The "Normalized Intensity" scale bar applies to all time-dependent SERS spectra. B) Relative intensity of Raman peak at 1438 cm^{-1}, assigned to νNN in DMAB, as a function of irradiation time. DMAB formation reaction rate (inset), determined from the slope of the initial linear portion of SERS intensity−time profiles.

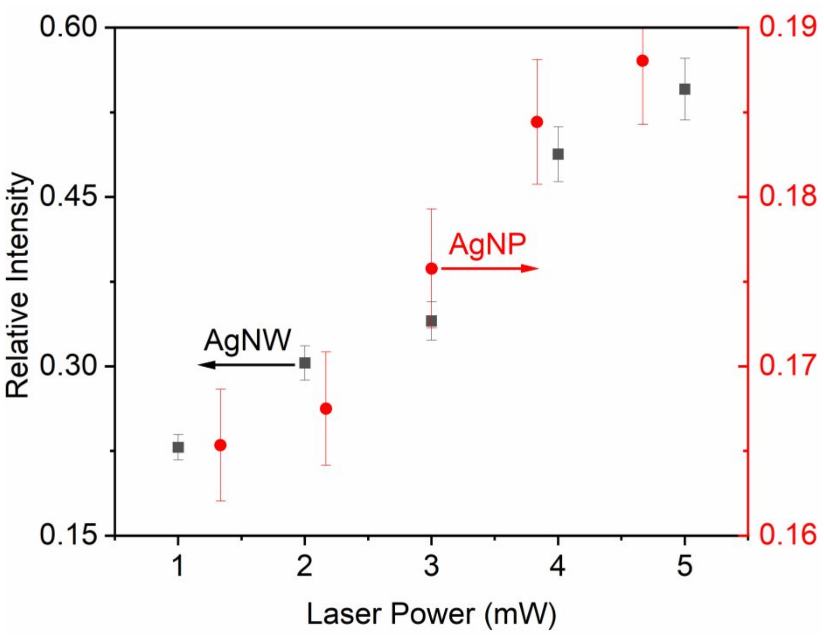

Figure S4. Relative intensity of Raman peak at 1590 cm^{-1}, assigned to νCC+δNH_2 PATP characteristic mode[1], as a function of laser power employed. Data points and y-axis in black represent the AgNWs, while data points and y-axis in red represent the AgNPs. PNTP adsorbed on AgNP and AgNW in HCl aqueous solution (pH 2).

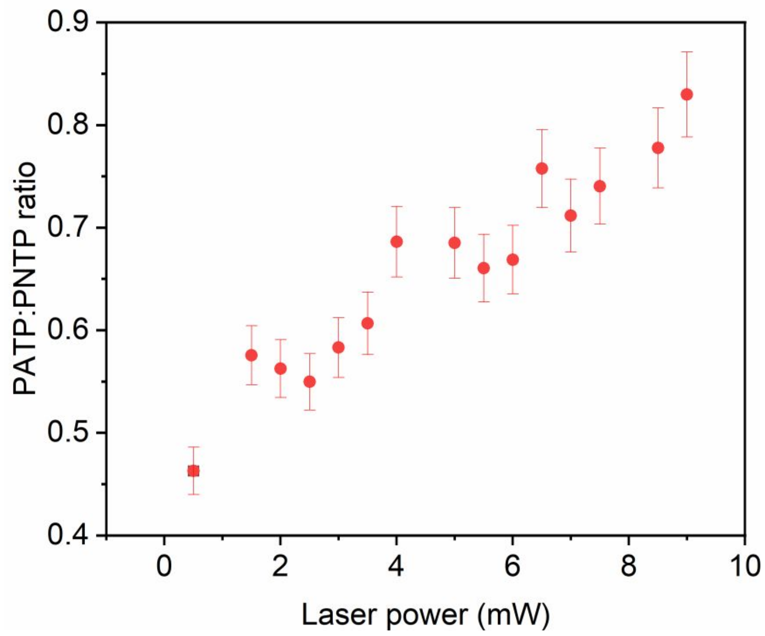

Figure S5: Obtained PATP/PNTP 1590/1338 cm^{-1} intensity ratios as a function of the laser power employed on the PNTP adsorbed on AgNW. The spectra were recorded under 633 nm excitation

References

(1) Huang, Y.-F.; Wu, D.-Y.; Zhu, H.-P.; Zhao, L.-B.; Liu, G.-K.; Ren, B.; Tian, Z.-Q. Surface-Enhanced Raman Spectroscopic Study of p-Aminothiophenol. *Physical Chemistry Chemical Physics* **2012**, *14* (24), 8485. https://doi.org/10.1039/c2cp40558j.
